# High confidence QTLs and key genes identified using Meta-QTL analysis for enhancing heat tolerance in chickpea (*Cicer arietinum* L.)

**DOI:** 10.3389/fpls.2023.1274759

**Published:** 2023-10-20

**Authors:** Raj Kumar, Vinay Kumar Sharma, Sagar Krushnaji Rangari, Uday Chand Jha, Aakash Sahu, Pronob J. Paul, Shreshth Gupta, Sunil S. Gangurde, Himabindu Kudapa, Reyazul Rouf Mir, Pooran M. Gaur, Rajeev K. Varshney, Dinakaran Elango, Mahendar Thudi

**Affiliations:** ^1^ Department of Agricultural Biotechnology and Molecular Biology, Dr. Rajendra Prasad Central Agricultural University (RPCAU), Pusa, Bihar, India; ^2^ Research Program-Accelerated Crop Improvement, International Crops Research Institute for the Semi-Arid Tropics (ICRISAT), Patancheru, Telangana, India; ^3^ Indian Council for Agricultural Research (ICAR)- Indian Institute of Pulses Research (IIPR), Kanpur, Uttar Pradesh, India; ^4^ Rice Breeding Innovations, International Rice Research Institute (IRRI), South Asia-Hub, Patancheru, Telangana, India; ^5^ Department of Plant Pathology, University of Georgia, Tifton, GA, United States; ^6^ Faculty of Agriculture, Sher-e-Kashmir University of Agricultural Sciences and Technology (SKUAST), Sopore, India; ^7^ Centre for Crop & Food Innovation, WA State Agricultural Biotechnology Centre, Food Futures Institute, Murdoch University, Murdoch, WA, Australia; ^8^ Department of Agronomy, Iowa State University, Ames, IA, United States; ^9^ Center for Crop Health, University of Southern Queensland, Toowoomba, QLD, Australia

**Keywords:** candidate genes, confidence interval, heat stress, Meta-QTLs, recombinant inbred lines

## Abstract

The rising global temperatures seriously threaten sustainable crop production, particularly the productivity and production of heat-sensitive crops like chickpeas. Multiple QTLs have been identified to enhance the heat stress tolerance in chickpeas, but their successful use in breeding programs remains limited. Towards this direction, we constructed a high-density genetic map spanning 2233.5 cM with 1069 markers. Using 138 QTLs reported earlier, we identified six Meta-QTL regions for heat tolerance whose confidence interval was reduced by 2.7-folds compared to the reported QTLs. Meta-QTLs identified on CaLG01 and CaLG06 harbor QTLs for important traits, including days to 50% flowering, days to maturity, days to flower initiation, days to pod initiation, number of filled pods, visual score, seed yield per plant, biological yield per plant, chlorophyll content, and harvest index. In addition, key genes identified in Meta-QTL regions like *Pollen receptor-like kinase 3 (CaPRK3), Flowering-promoting factor 1 (CaFPF1), Flowering Locus C (CaFLC), Heat stress transcription factor A-5 (CaHsfsA5)*, and *Pollen-specific leucine-rich repeat extensins (CaLRXs)* play an important role in regulating the flowering time, pollen germination, and growth. The consensus genomic regions, and the key genes reported in this study can be used in genomics-assisted breeding for enhancing heat tolerance and developing heat-resilient chickpea cultivars.

## Introduction

Heat stress is becoming a significant obstacle in achieving sustainable agricultural production in the context of global climate change, putting food security at risk. According to predictions of the Inter-governmental Panel on Climate Change (IPCC), the global average temperature rise of 0.2°C per decade may reach up to 1.5°C between 2030 and 2052 (https://www.bbc.com/news/newsbeat-4894757). The cool season legumes like pea (*Pisum sativum* L.), lentil (*Lens culinaris* Medik.), faba bean (*Vicia faba* L.), and chickpea (*Cicer arietinum* L.) are more affected by the heat stress compared to warm season legumes (Sita et al., 2017). Small holder farmers in the arid and semi-arid regions across the globe cultivate chickpea on the residual soil moisture in over 50 countries (Roorkiwal et al., 2020). According to FAOSTAT (2020), chickpea is the second most important legume crop in the world, after dry beans, with an area under cultivation of 14.5 million hectares and an annual production of 14.7 million tonnes and average seed yield of 1.014 tonnes ha-1. Reproductive stage heat stress adversely affects the production and productivity of chickpea, and average temperatures of 20-28°C are optimal for chickpea production (Devasirvatham et al., 2012). During critical stages of chickpea growth, like flower initiation and pod filling, temperatures over 32°C result in flower drop, pollen sterility, and pod abortion and thus lead to significant yield losses (Kaushal et al., 2013; Gaur et al., 2019; Devi et al., 2022). A brief exposure to chickpea over critical limits especially during the reproductive phase leads to irreversible damage (Kalra et al., 2008; Jha et al., 2021). Furthermore, it has been observed that temperatures ≥35°C under field conditions resulted in yield losses of up to 39% (Devasirvatham et al., 2015). However, the severity of heat stress depends on its intensity, temperature, and the process impacted by the crops.

An in-depth understanding of genetic variability is a pre-requisite for trait improvement. Towards this direction, several efforts were made to understand the genetic variability among the germplasm of chickpea. For instance, a large-scale variation for heat stress tolerance among the chickpea reference set genotypes was reported by Krishnamurthy et al. (2011). Further, in a set of 35 early maturing genotypes assessed for their sensitivity to heat stress, the genotypes ICC 13124, ICC 14284, ICC 14368, and ICC 14653 were reported to be heat stress tolerant and high-yielding (Upadhyaya et al., 2011). Most recently, 39 chickpea genotypes grown in normal-sown and late-sown environments were assessed for their response in relation to influence on seven physiological and four yield and yield-related traits. As a result, it was reported that GNG 1969, GNG 1488, PantG 186, RSG 888, CSJ 315, and GNG 1499 genotypes can be used as donors for enhancing heat tolerance in chickpea cultivars (Devi et al., 2022). Genetic variability was also evaluated among the recombinant inbred line (RIL) populations (Paul et al., 2018a; Kushwah et al., 2021a). A set of 121 genotypes was evaluated at two different locations (India and Ethiopia) and the genetic relationships were assessed at both phenotypic and genotypic levels (Getahun et al., 2021).

Besides assessment of the genetic variability in respect of tolerance to heat stress, some efforts were also made to identify the genomic regions responsible for heat tolerance. QTLs for number of filled pods, total number of seeds per plot, grain yield per plot and % pod setting were reported using RIL population developed from ICC 4567 (heat sensitive) × ICC 15614 (heat tolerant) (Paul et al., 2018b). Using SSR markers, QTLs for primary branch number and chlorophyll content were reported in F_2_ population derived from DCP 92-3 × ICCV 92944 (Jha et al., 2019). Additionally, Kushwah et al. (2021b) reported QTLs for heat tolerance using an inter-specific RIL population derived from the cross GPF 2 (heat tolerant) × ILWC 292 (heat sensitive). The related traits associated with QTLs are days to germination, days to flower initiation, days to 50% flowering, days to 100% flowering, plant height, grain yield, and membrane permeability index. Nevertheless, major QTLs for heat tolerance related traits were also reported using RIL population derived from DCP 92-3 (heat sensitive) and ICCV 92944 (heat tolerant). In addition, to QTLs, markers associated with the trait were also reported (Thudi et al., 2014; Varshney et al., 2019). However, only a few QTLs have been validated for their utilization in marker-assisted selection (MAS).

The Meta-QTL approach plays a pivotal role in precisely identifying stable QTLs across multiple studies for highlighting the consistency of location and effect for different QTLs of the same trait. The Meta-QTL analysis approach developed by Goffinet and Gerber (2000) can assist in narrowing down QTL regions with the of stringent selection of precise QTL (Soriano et al., 2021). Ultimately, it determines the “actual” number of QTLs affecting a trait and estimate their “actual” positions in the genome. Meta-QTL analysis has been applied to many cereals (maize, Kaur et al., 2021; rice, Sandhu et al., 2021; wheat, Ma et al., 2022) and legumes (pea, Klein et al., 2020; common bean, Arriagada et al., 2023), but not used for heat tolerance related traits in chickpea till now. In this study, we reported the identification of consensus genomic regions and associated key genes deploying Meta-QTL analyses. Consequently, the identified Meta-QTLs and associated key genes can be used for developing heat stress tolerant chickpea varieties.

## Materials and methods

### Compilation of reported QTLs for heat tolerance

All the QTLs associated with heat stress tolerance related traits were retrieved from four independent studies reported between 2018 to 2021. The reported QTLs were associated with 28 different heat stress tolerance related traits. The QTLs were re-grouped into four major trait categories (i) morphological (primary branches number, PB; plant height, PH), (ii) phenological (days to flower initiation, DFI; days to maturity, DM; days to 50% flowering, DFF; days to 100% flowering, DHF; and pollen viability, PV) (iii) Physiological (days to germination, DG; chlorophyll content, CHL; nitrogen balance index, NBI; cell membrane stability, CMS; normalized difference vegetation index, NDVI; membrane permeability index, MPI and relative leaf water content, RLWC) and (iv) yield and yield related traits (days to pod initiation, DPI; days to pod formation, DPF; number of filled pods, FPod; 100 seed weight, 100SW; seed yield per plant, SYPP; biological yield per plant, BYPP; harvest index, HI; pod setting percentage, % Podset; total number of seeds per plot, TS; grain yield, GY; visual score, VS; number of pods per plant, NPP; biomass, BIO and yield, YLD). Among 138 reported QTLs, 59 major (PVE ≥ 10%) and 79 minor QTLs (PVE < 10%) were categorized. We compiled the information on (i) population type and size of the mapping population (ii) QTL ID with closely linked flanking markers (iii) LOD score (iv) phenotypic variation explained (PVE) or R^2^ value (v) position of the peak and associated confidence interval (CI). The missing CI values were calculated by using two different equations proposed by Darvasi and Soller (1997) for different mapping populations. The formula used for F_2_ and BC population is CI = 530/N × R^2^ but for RILs CI = 163/N × R^2^, wherein N denotes size of mapping population and R^2^ denotes PVE for each QTL; the numerical value 530 and 163 are the population-specific constants obtained from different simulation. If the peak position was missing in any case, the mean of two flanking markers position was considered as the QTL peak.

### Construction of consensus map

Consensus map was constructed by using four genetic maps from different studies to represent all markers associated with QTLs in a single map with the help of R package based LPMerge algorithm (Endelman and Plomion, 2014). LP merge in R constructs consensus maps using the root mean square error (RMSE) value, which is based on the linear programming method. In order to determine the optimal consensus map, the model with the least map size and least RMSE value was chosen and accepted as a final map for performing Meta-QTL analysis. Ultimately, the dense consensus map was constructed based on F_2_ and RIL populations using 1771 markers.

### QTL projection

The QTLs explaining at least 10% of phenotypic variation for the target trait were projected on the consensus map using BioMercator Version 4.2.3 (Arcade et al., 2004), and associated information for each QTL such as confidence interval (95%), peak position, LOD score and PVE (Sosnowski et al., 2012). In order to project QTLs, only the QTLs having the same flanking markers in the consensus map were used (Soriano et al., 2021).

### Meta-QTL analysis

As a result of the projection, Meta-QTL analyses were performed on QTL cluster for each chromosome individually using the two-step algorithm proposed by Veyrieras et al. (2007) in BioMercator Version 4.2.3 (Arcade et al., 2004). In the first step, the best Meta-QTL model was selected out of many models available in the software by using selection criteria as Akaike information criterion (AIC), AIC corrected (AICc), AIC model 3 (AIC3), Bayesian information criterion (BIC), and Average weight of evidence (AWE). In order to select a Meta-QTL model, at least three models must achieve the lowest values of the selection criteria. In the second step, meta-QTLs were generated using best Meta-QTL model, which ensures that the number is generally less than the number of projected QTLs. While performing QTL meta-analysis, it is necessary to have independent QTLs for the same trait from different mapping populations, different locations, and different environmental conditions (Goffinet and Gerber, 2000).

### Detecting candidate genes underlying the Meta-QTL region

In order to retrieve the candidate genes in Meta-QTL regions, the physical position of flanking markers was used as an input using the https://cegresources.icrisat.org database with the help of chickpea assembly (Varshney et al., 2013). The function associated with each gene was determined as per Thudi et al. (2021).

## Results and discussion

Heat stress is one of the major constraints among abiotic stresses in chickpea. During the flowering stage, elevated temperatures (> 35°C) hamper pollen germination on the stigma and tube growth inside style, preventing it from reaching up to female gametes, and thus it leads to flower drop, pod abortion or abnormal pod setting (Devasirvatham et al., 2012; Devasirvatham et al., 2015; Devasirvatham and Tan, 2018). Efforts were made to identify the heat tolerant germplasm as well as map the genomic regions using both linkage mapping (Paul et al., 2018b; Jha et al., 2019 and Jha et al., 2021; Kushwah et al., 2021b) and linkage disequilibrium mapping (Thudi et al., 2014; Varshney et al., 2019). Nevertheless, the genomic regions reported in different studies were large for use in genomics-assisted breeding programs. To reduce the genomic intervals and identify the consensus genomics regions for heat tolerance, we have used Meta-QTL analysis approach in this study.

### QTLs identified for heat stress tolerance

In case of chickpea, to date, a total of four independent studies reported QTLs for heat tolerance related traits. Among these studies, two studies used SNPs based on genotyping by sequencing approach, one study used SNP based on double digest restriction-site associated DNA sequencing approach while the other study used SSR markers. Among three RIL populations, two were intra-specific (ICC 4567 × ICC 15614, DCP 92-3 × ICCV 92944) and another one was inter-specific (GPF 2 × ILWC 292). Using advanced generations of DCP 92-3 × ICCV 92944, QTLs were mapped at F_2_ and F_7_ stages by Jha and colleagues in 2019 and 2021 respectively. The size of the mapping populations ranged from 184 to 292 lines (**Table 1**). In total, 138 QTLs were reported earlier for 28 different traits. Further, among 138 QTLs reported earlier, 59 and 79 were major (≥10% phenotypic variation explained, PVE) and minor QTLs (<10% PVE), respectively. The confidence intervals of these QTLs ranged from 2 - 44.29 cM with an average of 9.2 cM (**Supplementary Table 1**).

**Table 1:** Summary of QTLs for heat tolerance related traits reported between 2018-21.

Mapping population	Population type	Population size	Number and type of markers	Traits	QTLs	Reference
ICC 4567 × ICC 15614	RILs (F_8-9_)	292	271 SNPs	6	8	Paul et al., 2018b
DCP 92-3 × ICCV 92944	F_2_	206	39 SSRs	2	2	Jha et al., 2019
DCP 92-3 × ICCV 92944	RILs (F_7_)	184	788 SNPs	12	77	Jha et al., 2021
GPF 2 × ILWC 292	RILs (F_8_)	187	673 SNPs	12	51	Kushwah et al., 2021b

### QTLs on the consensus map

For projection of all QTLs, a dense consensus map comprising of 1069 markers spanning a total genetic distance of 2233.5 cM was constructed. Nevertheless, individual genetic maps reported 39 (Jha et al., 2019) to 788 markers (Jha et al., 2021). The length of each linkage group varied between 68.1- 497.7 cM, with an average distance of 279.19 cM (**Supplementary Table 2**). Compared to Kushwah et al. (2021a; 2021b) genetic map (4569.09 cM), a reduction of 2.04 folds genetic distance was observed in the consensus map developed in the present study. Drastic reduction in the genetic distance of consensus map indicated the robustness of consensus map for identification of more precise Meta-QTLs. The number of markers per linkage group ranged from 68 (on CaLG08) to 253 (on CaLG06). The densest map was observed for the linkage group CaLG08 with a marker density of 0.9 marker/cM. Amongst 138 QTLs, 65.21% (90) QTLs could be projected on consensus genetic map we developed. This could be due to lack of common markers for mapping all the QTLs.

### Meta-QTLs identified

**Figure 1:**
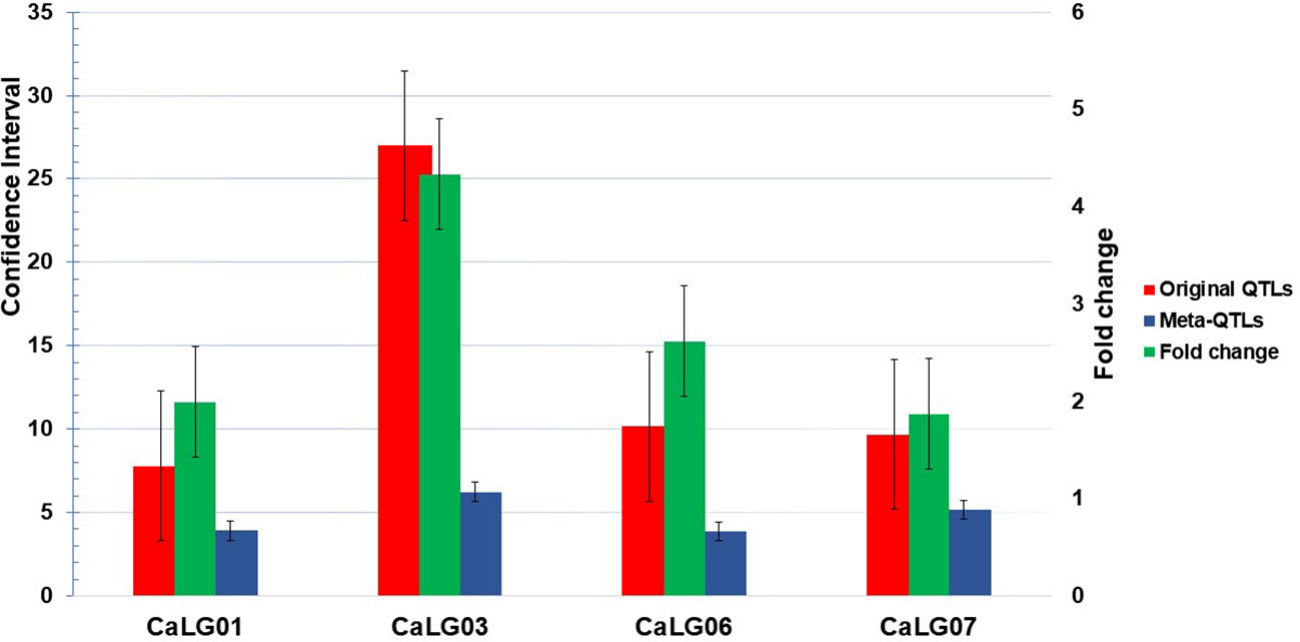
Comparison of confidence interval (CI) between original QTL and Meta-QTL; CaLG03 shown maximum reduction in CI (four times against original QTL) but CaLG07 shown minimum reduction in CI.

**Figure 2:**
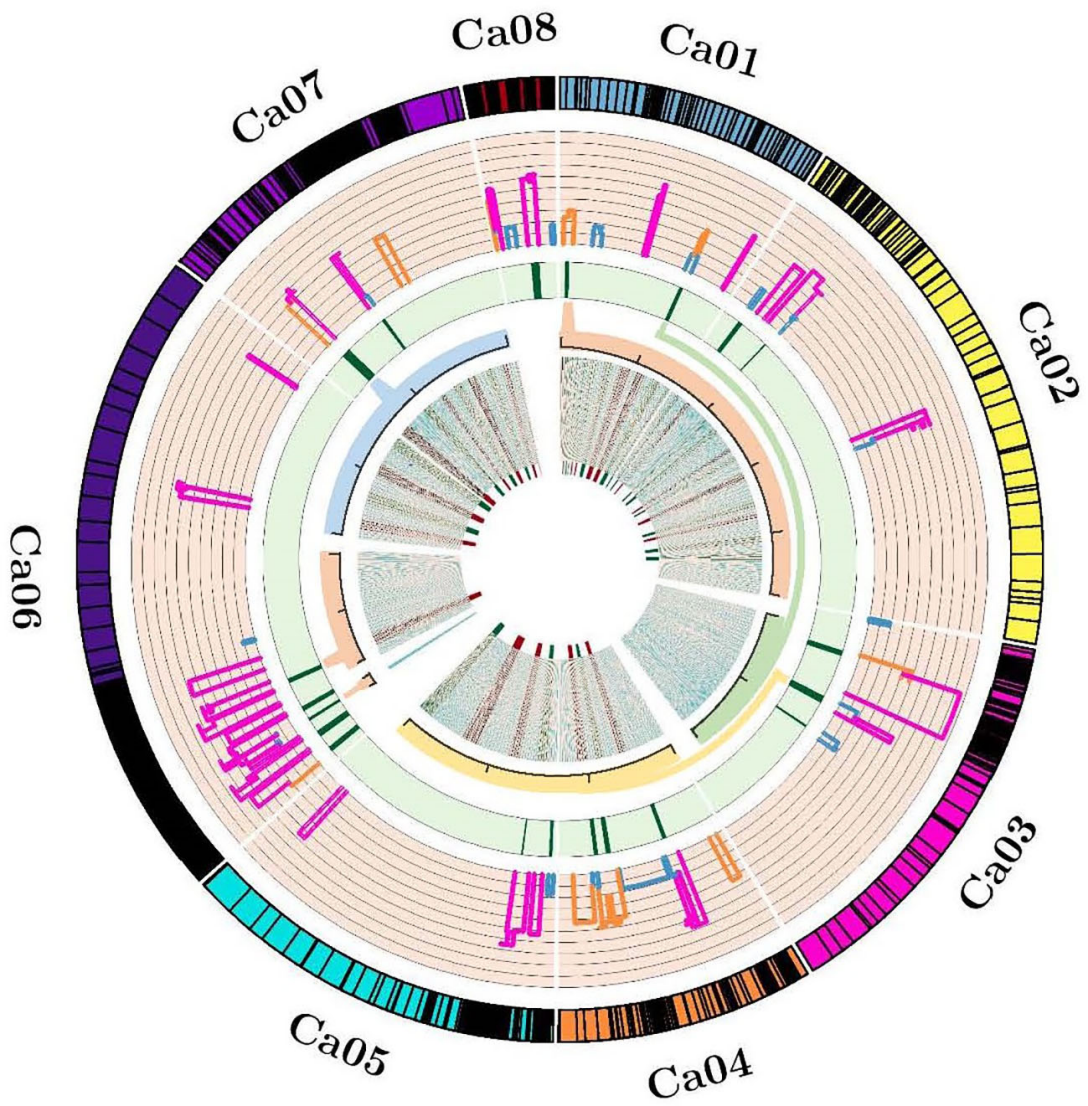
Circos indicating the projected QTLs, Meta-QTLs and genes. ***From outside to inside***, Track 1 representing ideogram of linkage groups and black bands are showing marker density; Track 2 representing the histogram of projected QTLs and width of histogram is showing the confidence interval; Track 3 representing heatmap of identified Meta-QTLs and width of heatmap is indicating the reduction in confidence interval of Meta-QTLs; Track 4 representing magnified view of accepted Meta-QTL regions into the redundant physical interval; Track 5 representing the density map of identified candidate genes.

Of 90 QTLs projected onto the consensus map, 22 were grouped into six Meta-QTL regions, comprising QTLs from at least two different studies (**Table 2**; **Figure 1**). The remaining 68 QTLs were either singletons or had a very high confidence interval, and for different traits of same study which could not be considered as Meta-QTL by the Meta-QTL clustering algorithm. The six Meta-QTLs were founded, and distributed on four linkage groups of chickpea (CaLG01, CaLG03, CaLG06, and CaLG07; **Figure 2**). A maximum of two (on CaLG01 and CaLG06) and a minimum of one (on CaLG03 and CaLG07) Meta-QTLs were identified on linkage groups. The number of QTLs in each Meta-QTL varied from two to six (**Table 2**). Interestingly, Meta-QTL4 contained six QTLs clustered for four different traits (FPOD, VS, SYPP and BYPP). Similarly, Meta-QTL5 contained five QTLs clustered for three different traits (CHL, BYPP and HI). The average number of QTLs in each Meta-QTL regions was 3.66 QTLs per Meta-QTL. The confidence interval at 95% of each Meta-QTL ranged from 2.97 cM for Meta-QTL4 on CaLG06 to 6.23 cM for Meta-QTL3 on CaLG03. The average confidence interval for the Meta-QTL was 4.49 cM. The Meta-QTLs identified from the QTL clusters were found to have significantly low confidence interval compared to the original projected QTLs. A graphical representation of CI of Meta-QTL and original QTL on each of the chickpea chromosome has been presented in **Figure 2**. The average CI fold reduction of Meta-QTL compared to original QTLs was computed to be 2.7 folds. These results signify that the Meta-QTLs identified in the present study are highly stable and have reduced confidence interval; thereby enhancing the detection of candidate genes and providing high mapping resolution.

**Table 2:** Details of Meta-QTLs identified for heat tolerance related traits in chickpea.

Meta-QTL	Linkage Group	Position			CI (95%)	Flanking markers	No. of QTLs	Traits^†^
		Absolute	CI start	CI end				
Meta-QTL1	CaLG01	9.46	7.575	11.345	3.77	SCA1_34969214 - SCA1_18234886	2	DM, DFF
Meta-QTL2	CaLG01	152.9	150.885	154.915	4.03	SCA1_19787122 - SCA1_19586410	4	DFI, DM (2), DPI
Meta-QTL3	CaLG03	48.8	45.685	51.915	6.23	TA142 - NCPGR149	2	PB, NDVI
Meta-QTL4	CaLG06	58.31	56.825	59.795	2.97	SCA6_10670773 - SCA6_10672468	6	FPOD, VS, SYPP (2), BYPP (2)
Meta-QTL5	CaLG06	110.96	108.565	113.355	4.79	SCA6_7820481 - SCA6_7929338	5	CHL, HI, BYPP (3)
Meta-QTL6	CaLG07	36.96	31.8	42.12	5.16	SCA7_28235343 - SCA7_36854126	3	HI, RLWC, NBI
Average					4.49		3.66	

**^†^** Days to maturity (DM); Days to 50% flowering (DFF); Days to flower initiation (DFI); Days to pod initiation (DPI); Primary branch number (PB); Normalized difference vegetation index (NDVI); Number of filled pods per plot (FPOD); Visual score (VS); Seed yield/plant (SYPP); Biological yield/plant (BYPP); Chlorophyll content (CHL); Harvest index (HI); Relative leaf water content (RLWC); Nitrogen balance index (NBI).

### Key genes in Meta-QTL regions

We identified a total of 1346 genes among 6 Meta-QTL regions (**Supplementary Table 3**). On annotation, 55 genes belonged to a wide range of gene-families that are directly or indirectly related to abiotic stress tolerance especially heat tolerance (**Table 3**) and 1291 genes were either putative or uncharacterized. Among 6 Meta-QTL regions, the Meta-QTL6 on CaLG07 harbored maximum number of genes (24 genes) with known functions followed by Meta-QTL1 on CaLG01 (23 genes), Meta-QTL3 on CaLG03 (7 genes) and Meta-QTL4 on CaLG06 harbored single gene with known function (**Table 3**). A total of 11, six, four and one genes were found to code for ethylene responsive transcription factor, leucine rich repeat extension like protein, DnaJ heat shock protein and pollen receptor kinase respectively. Alternatively, some genes encoding proteins like peroxidase and superoxidase dismutase have a role in defying oxidative stress and recovering plants from heat stress damage (**Table 3**).

**Table 3:** Summary of key genes underlying in Meta-QTLs regions with known functions.

Meta-QTL ID	Gene ID	Predicted Function
Meta-QTL1	Ca_14718	Flowering-promoting factor-like protein
	Ca_24217	Pollen-specific leucine-rich repeat extensin-like protein 1
	Ca_24225	Pollen-specific leucine-rich repeat extensin-like protein 1
	Ca_17354	Ethylene response factor
	Ca_22117	Heat shock-like protein, putative
	Ca_19297	Ethylene-responsive transcription factor ERF017-like
	Ca_19296	Ethylene-responsive transcription factor ERF017-like
	Ca_19295	Ethylene-responsive transcription factor erf017-like protein
	Ca_18344	Pollen-specific leucine-rich repeat extensin-like protein 2
	Ca_18341	Heat shock protein
	Ca_18590	Heat shock protein
	Ca_20281	Peroxidase 7-like
	Ca_18530	MADS-box protein FLOWERING LOCUS C-like
	Ca_22571	Zinc finger protein CONSTANS-LIKE 9-like
	Ca_22561	Chaperone dnaj-domain protein, putative
	Ca_22560	Pollen-specific leucine-rich repeat extensin-like protein 1
	Ca_24386	F-box protein
	Ca_19442	Pollen-specific leucine-rich repeat extensin-like protein 1
	Ca_19459	F-box protein SKIP2
	Ca_19466	Putative F-box protein At3g16210
	Ca_25480	Pollen-specific leucine-rich repeat extensin-like protein 1
	Ca_22107	Calcium-transporting atpase 8, plasma membrane-type
	Ca_18324	Dnaj protein ERDJ2A-like
Meta-QTL3	Ca_06050	Receptor protein kinase-like protein
	Ca_06047	Receptor protein kinase-like protein
	Ca_06034	Ethylene-responsive element binding protein 1
	Ca_06032	Ethylene-responsive element binding protein 1
	Ca_06021	Ser/Thr protein kinase
	Ca_06019	Ser/Thr protein kinase
	Ca_05985	Zinc finger protein CONSTANS-like protein
Meta-QTL4	Ca_09583	Putative ETHYLENE INSENSITIVE 3-like 4 protein
Meta-QTL6	Ca_18924	Heat shock protein
	Ca_18948	Ethylene response factor
	Ca_11765	Ethylene-responsive transcription factor CRF1-like
	Ca_10067	Calmodulin-binding family protein
	Ca_10013	Pollen-specific leucine-rich repeat extensin-like protein 3
	Ca_16155	Pollen receptor-like kinase 3
	Ca_16156	F-box protein SKP2A-like
	Ca_16168	COP1-interacting-like protein
	Ca_16170	Dnaj homolog subfamily B member 6-like isoform X3
	Ca_16171	Dnaj homolog subfamily B member 6-like isoform X3
	Ca_16180	Ethylene response factor
	Ca_17621	Calcium-binding protein KIC
	Ca_17627	Myb transcription factor
	Ca_17638	Ethylene-responsive transcription factor ERF023-like
	Ca_17639	ABA-inducible bhlh-type transcription factor
	Ca_17762	Late embryogenesis abundant protein
	Ca_17776	F-box/kelch-repeat plant protein
	Ca_17780	F-box/kelch-repeat protein At3g61590-like
	Ca_17789	F-box/LRR-repeat protein 12
	Ca_17791	F-box protein interaction domain protein
	Ca_13731	Calmodulin-binding receptor-like cytoplasmic kinase
	Ca_18949	Dnaj-class molecular chaperone
	Ca_13761	Calmodulin-binding heat-shock protein
	Ca_13713	Myb transcription factor

In chickpea, completion of the process of pollen germination and growth of the pollen tube is essential for successful fertilization. In Meta-QTL1, five genes namely Ca_24217, Ca_24225, Ca_22560, Ca_19442, and Ca_25480 encode for pollen-specific leucine-rich repeat extensin-like protein 1 and the gene Ca_18344 codes for pollen-specific leucine-rich repeat extensin-like protein 2. While in case of Meta-QTL6 the gene Ca_10013 codes for pollen-specific leucine-rich repeat extensin-like protein 3. Pollen-specific leucine-rich repeat extensin (LRXs), harbor a leucine-rich repeat domain and an extensin domain, are essential for pollen germination and growth in Arabidopsis (Wang et al., 2018). In addition, like the *LePRK2* gene in tomato (*Solanum lycopersicum*), we identified a gene (Ca_16155; referred as *CaPRK2;* Ca stands for *Cicer arietinum*) that encodes pollen receptor kinase, which has been implicated in signaling during pollen germination and tube growth as well as in mediating pollen (tube)-pistil communication. In case of tomato, it was demonstrated that *LePRK2* positively regulates pollen germination and tube growth (Zhang et al., 2008).

We also identified important genes like Flowering-promoting factor 1-like protein 1 (Ca_14718; referred as *CaFPF1*) and MADS-box protein FLOWERING LOCUS C (Ca_18530; referred as *CaFLC*) in Meta-QTL1 genomic region. In addition, we identified a gene Ca_14718 that encodes *FPF1* (*Flowering-promoting factor 1*) reported to be involved in the genetic control of flowering time in plants. For instance, in case of rice, it was demonstrated that the *OsFPFL4* gene was involved in modulating the root and flower development by affecting auxin and ROS homeostasis (Guo et al., 2020). However, MADS-box transcription factors (TFs), *FLOWERING LOCUS C (FLC)* along with *SHORT VEGETATIVE PHASE (SVP)* have been reported to form a complex to repress the expression of genes that initiate flowering in Arabidopsis (Mateos et al., 2015). This indicates that the Meta-QTL1 harbor genes that control both flower initiation as well as repression of the flowering. In general, the genotypes with early flowering feature record higher yield by escaping the reproductive stage heat stress as well as end season drought (Manchikatla et al., 2021). In Meta-QTL1 and 6, we identified genes (Ca_18341, Ca_18590 and Ca_18924), that code for heat shock proteins or heat stress transcription factor A-5 referred as *CaHsfsA5*. Among 20 heat stress transcription factors (*Hsfs*), *Hsfs A4* and *A5* form a group with distinguished gene activity as *A4 Hsfs* are potent activators of heat stress gene expression, whereas *A5 Hsfs* act as specific repressor of *HsfA4* activity (Baniwal et al., 2007). Another gene, referred as *CaERN3* that encodes ethylene-responsive transcription factor *ERN3* was reported to repress the *ERN1/ERN2*-dependent transcription activation in legumes. This Meta-QTL region habors QTLs for NBI and CHL. Reduction of nitrogen balance index and chlorophyll content is the common phenomenon in plants and it was reported in maize during post flowering heat stress (Bheemanahalli et al., 2022).

## Conclusions

Meta-QTL analysis for the traits related to heat stress tolerance is an effective approach of unravelling the concise and precise QTLs from many QTLs reported earlier. In the present study, the CI of the identified Meta-QTLs is reduced by 2.7 folds against the original QTL CI. In addition, key genes like *Pollen receptor-like kinase 3* (*CaPRK3), Flowering-promoting factor 1 (CaFPF1), FLOWERING LOCUS C (CaFLC), Heat stress transcription factor A-5* (*CaHsfsA5*), and *Pollen-specific leucine-rich repeat extensin (LRXs)*, identified in the Meta-QTL regions are known to play key role in regulating the flowering, pollen germination and growth etc, which can be further explored in detail. Large-scale germplasm sequence information (Thudi et al., 2016a; Thudi et al., 2016b; Varshney et al., 2019; Varshney et al., 2021; Thudi et al., 2023) and phenotypic data in public domain can be used for haplo-pheno analysis and identify superior haplotypes for use in heat tolerance breeding. The Meta-QTLs with reduced confidence interval as well as key genes reported in the study can be used in chickpea breeding programs for developing heat resilient chickpea varieties.

## Data availability statement

The original contributions presented in the study are included in the article/**Supplementary Material**. Further inquiries can be directed to the corresponding authors.

## Author contributions

RK: Data curation, Methodology, Writing – original draft. VKS: Resources, Writing – review & editing. SKR: Formal Analysis, Methodology, Writing – review & editing. UCJ: Resources, Writing – review & editing. AS: Formal Analysis, Writing – review & editing. PJP: Resources, Writing – review & editing. SG: Formal Analysis, Methodology, Writing – review & editing. SSG: Resources, Writing – review & editing. HK: Resources, Writing – review & editing. RRM: Resources, Writing – review & editing. PMG: Resources, Writing – review & editing. RKV: Resources, Writing – review & editing. DE: Writing – review & editing. MT: Conceptualization, Supervision, Writing – review & editing.

## Supplementary material

Supplementary Table 2 Marker order and their position on the consensus genetic map developed using four genetic mapping studies.
